# Improving the Standard for Deep Brain Stimulation Therapy: Target Structures and Feedback Signals for Adaptive Stimulation. Current Perspectives and Future Directions

**DOI:** 10.7759/cureus.2468

**Published:** 2018-04-12

**Authors:** Franz Hell, Thomas Köglsperger, Jan Mehrkens, Kai Boetzel

**Affiliations:** 1 Neurology, Ludwigs-Maximilians-University Munich, Munich, DEU; 2 Department of Neurology, Ludwigs-Maximilians-University Munich, Munich, DEU; 3 Department of Neurosurgery (head of Functional Neurosurgery), Ludwigs-Maximilians-University Munich, Munich, DEU

**Keywords:** dbs target, adaptive dbs, machine learning

## Abstract

Deep brain stimulation (DBS) is an established therapeutic option for the treatment of various neurological disorders and has been used successfully in movement disorders for over 25 years. However, the standard stimulation schemes have not changed substantially. Two major points of interest for the further development of DBS are target-structures and novel adaptive stimulation techniques integrating feedback signals. We describe recent research results on target structures and on neural and behavioural feedback signals for adaptive deep brain stimulation (aDBS), as well as outline future directions.

## Introduction and background

Deep brain stimulation (DBS) is an established option for the treatment of movement disorders, including essential tremor, Parkinson’s disease and dystonia, and other neurological disorders, such as epilepsy and neuropathic pain. It also is being investigated for psychiatric disorders, i.e., depression, obsessive-compulsive disorder, and Tourette syndrome. DBS has been used successfully in movement disorders for over 25 years; however, the technology has evolved only very slowly and the stimulation scheme (non-adaptive constant stimulation) has not changed at all. To advance DBS technology, two major points of interest are target-structures and novel adaptive stimulation techniques integrating feedback signals [1].

## Review

Refined targeting approaches for DBS

An important research area is the definition of brain structures involved in the pathophysiology of the disease to be treated with DBS. Targeting of specific (sub)-structures and fibres involved in the generation of pathological neural activity and avoiding others will be imperative to improve the clinical DBS effect and limiting side-effects. Currently, there are a handful of FDA-approved targets, including the internal segment of the globus pallidus (GPi), nucleus ventralis intermedius (ViM), subthalamic nucleus (STN), and several other investigational targets, used for DBS in movement and other neurological disorders (often more than one for a specific symptom) [2]. New programming approaches, such as current steering [3], already allow manipulating the volume of tissue activated, and therefore, a more specific stimulation of neural structures and future miniature implants [4] will push this boundary even further.

So far, surgical planning is commonly done based on basic structural MR images and programming is still dependent on trial-and-error guided programming. To better understand the effects of stimulation and guide programming, it is important to refine our theory of neural circuit wiring and their function during normal behaviour and dysfunction in disease. Current research in humans highlights different network structures connected to individual DBS targets and explores oscillatory mechanisms [5] involved in the generation of the pathophysiology of respective symptoms.

Research in dystonia patients has shown that stimulation of the ventral GPi is more efficient in alleviating dystonic symptoms [6]. Rozanski and colleagues used diffusion tensor tractography to study the connectivity patterns of different target structures and specific DBS electrode locations; they reported considerable differences in connectivity profiles of the ventral and dorsal GPi. The authors interpreted their results in favor of different functional subsystems in the ventral and dorsal GPi and recommended that targeting specific areas could play an important role in promoting clinical DBS effects in dystonia [7].

The ViM is a popular target for DBS in medically intractable tremors, like the Parkinsonian or essential tremor. Tractography studies show structural connectivity between ViM and motor cortical, subcortical, cerebellar sites, and the brainstem [8]. Cagnan and colleagues compared subthalamic stimulation near tremor frequency in Parkinson's disease and stimulation of the ventrolateral thalamus in essential tremor, describing the differences in the response of the behavioural tremor characteristics [9]. The authors reasoned that different networks might be involved in Parkinsonian rest and essential tremor. Several research groups showed the involvement of the dentato-rubro-thalamic tract in the subthalamic region in tremor control [10] and reported successful guidance of DBS surgery with fibre tracking techniques [11].

According to reports, PD patients showed similar improvement in motor function after pallidal as well as subthalamic stimulation [12], while others stated that STN DBS was superior in improving off-drug phase motor symptoms and functioning [13]. Several research groups pointed towards the posterior lateral part of the STN as the preferred DBS target region [1]. The STN was reported to be grouped into a posterolateral motor and a gradually overlapping central associative area, while the limbic area was reported in the anteromedial part of the nucleus [14-16]. Accola et al. used subthalamic local field potential recordings from PD patients to investigate the relation between oscillatory activity, mainly beta activity, and subthalamic fibre connectivity. The area of the STN with the highest beta power, the dorsolateral portion, predominantly projected to the motor, premotor, and also to the limbic and associative areas. Ventral areas associated with connectivity to the medial temporal regions (e.g., amygdala and hippocampus) [5]. It has been shown before that beta power correlates with bradykinesia and rigidity [17-18]. Different research groups [19-23] suggest that the posterior lateral subthalamic area with the most beta activity might be a sweet spot to guide DBS electrode placement. Several groups also report that DBS of the medial and limbic STN can result in the stimulation of the medial forebrain bundle and can induce side effects, such as hypomania [20, 24].

Understanding the functionality of different target structures and their network connectivity is imperative to further improve targeting. At this time, there is an ongoing debate about the real parcellation of the STN, its connectivity, and functional relevance of different subsystems [25]. To a degree, this can be said for every major DBS target [26-27]. Advances in structural imaging methods, such as ultra-high field MRI and novel data analysis algorithms, inspired by machine learning approaches, such as deep learning [28-29], will ultimately refine our understanding and conception of different neural structures and their wiring in health and disease. New stimulation techniques, such as non-invasive deep brain stimulation via temporally interfering electric fields [30], and advances in functional brain imaging and analysis methods [31-32] could provide a novel way to find and elaborate target structures and help individualize surgery planning and stimulation. To further advance our understanding, the study of network dynamics in humans and animal models during behaviour and their relation to the pathophysiology of the disorder as well as the manipulation of neural circuits with methods, such as electric or optogenetic stimulation, will provide further insights into the neural mechanisms, potential target structures, and effects of DBS.

New stimulation schemes for DBS

Neurostimulation systems available today provide stimulation in an open-loop manner, which means that stimulation settings are pre-programmed and do not automatically respond to changes in the patient’s clinical symptoms or in the underlying neural substrate. While open-loop stimulation paradigms remain state of the art, limitations (like overall efficiency or side-effects) have become more evident as clinical experience grows. Adjusting therapy remains time-consuming, requiring physicians to evaluate countless combinations of stimulation parameters to achieve the “best” therapy. Current DBS practice requires patients to follow-up for months postoperatively to optimize therapy. In the future, feedback signals will ideally be integrated into closed-loop stimulation systems that rapidly respond to real-time patient needs and obviate the need for human programming.

Ideally, patient and disease-specific biomarkers could help optimize and individualize therapy, help to find the optimal parameters for stimulation, and ultimately, close the loop. Local field potentials with their high temporal resolution, as well as spatial specificity, can easily be measured with DBS electrodes or implanted neural sensors and hold great promise as such biomarkers. Several oscillatory patterns in different structures, such as aberrant subcortical tremor and beta-frequency activity [17, 33-35], pathological cross-frequency coupling [36-37], or pathological coherence of neural activity between cortical and subcortical structures [38], have been reported to be correlated with clinical symptoms. Initial approaches incorporating local field potentials (LFP) as feedback signals into adaptive DBS using beta frequency amplitude power to trigger stimulation [39] could show clinical improvement of symptoms compared to standard DBS. A new approach by Tinkhauser et al. targeted potentially pathological beta bursts with long duration sparing, presumably functionally relevant short beta bursts, and could show a similar improvement [40]. Despite early success, challenges have yet to be overcome. Beta power in the STN, for example, correlates with rigidity and bradykinesia but not with tremor [41-42], which is linked to low-frequency activity at tremor frequency. PD patients, for example, often show multiple symptoms; a single one-dimensional biomarker might, therefore, be only partly useful. Most neural biomarkers (like elevated beta frequency oscillations) are not only correlated with disease symptoms but are also reactive to medication [43-44], are functionally relevant and modulated during normal behaviour (i.e., movement or cognition) [45-46], and evolve with disease progression.

**Figure 1:**
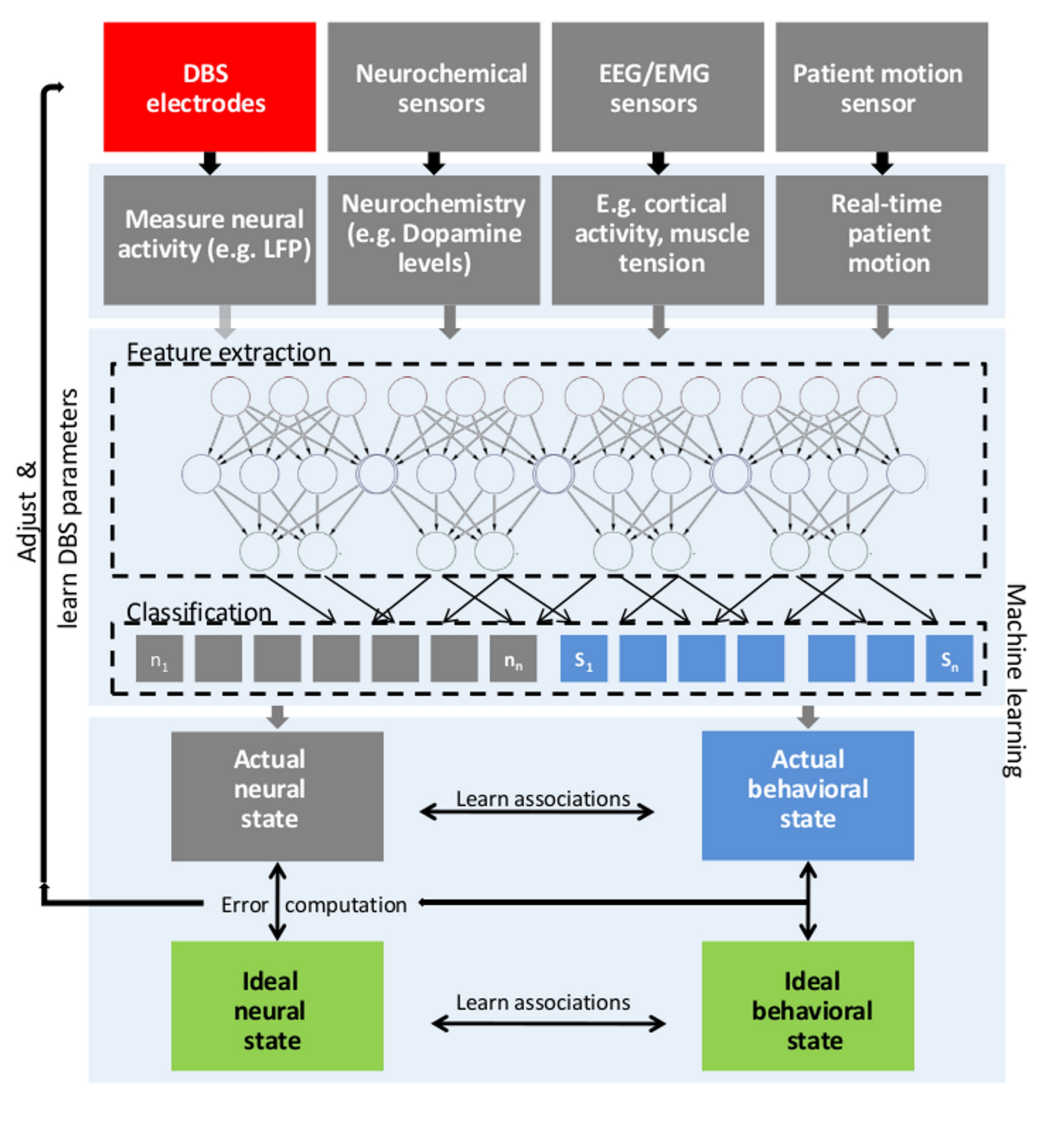
Schematic depiction of general aDBS framework General closed loop DBS for adaptive adjustment of deep brain stimulation (DBS) parameters based upon real time patient measurements, such as electrophysiological signals (LFP, M/EEG, EMG), neurochemical parameters and behavioural measurements and machine learning. First, features from different possible signal sources are learned (e.g. beta frequency amplitude, phase of tremor oscillations) using deep learning approaches to classify between different behavioural (clinical) states (e.g. bradykinesia, tremor) and corresponding neural states. Then, actual states are compared with ideal states and stimulation parameters are adjusted and finally learned via reinforcement learning. In this closed-loop scheme, the stimulation parameters are adjusted within clinical limits based upon the difference between actual neural/behavioural and desired neural/behavioural state. aDBS: adaptive deep brain stimulation; EEG: electroencephalography; EMG: electromyography; LFP: local field potentials

Body measurements during rest and behaviour (allowing for the assessment of behaviour and symptom severity) could be a promising alternative or additional feedback signal for use in aDBS. Kinematic parameters can be extracted from signals collected by inertial sensor units and then be used to quantify clinical symptoms, such as tremor, rigidity, or bradykinesia. Inertial sensor measurements have already been successfully used for adaptive stimulation in essential tremor [47]. Singh and colleagues suggested that gait parameters are affected by DBS surgery [48]. We propose that by integrating electrophysiological recordings and information from kinematic measurements and other sensors like electromyography, the state of the patient, the severity of disease symptoms, and related neural activity can be ultimately learned and classified [49]. As an example, it is conceivable that an algorithm can learn to extract features from neural recordings, such as aberrant beta frequency activity, and relate them to kinematic parameters describing bradykinesia or rigidity as measured during a simple movement task in a calibration setting (for a schematic depiction, see Figure 1). Stimulation parameters can then be varied within clinical limits, and those parameters that are associated with optimal behavioural and neural parameters could then be learned via reinforcement learning. Alternative stimulation protocols, steering of the volume of tissue activated (VTA) via segmented leads and parameters like stimulation intensity, frequency, temporal stimulation pattern [50], pulse duration, and timing of stimulation relative to neural activity could be tested and an optimal parameter setting ultimately be learned and adjusted, when needed, closing the loop. As a first step, it is important to establish approaches to guide current steering based on structural as well as functional imaging and behavioural assessment. A future direction could be to collect standardized kinematic and behavioural measurements, together with neural recordings and imaging on a large scale basis, during feedback-guided DBS programming to establish a comprehensive data basis. To establish a real-time link between behavioural and neural measurements, a data analysis model has to be able to extract features from both sources and reliably decode clinical symptom severity as well as find predictors for changes in behaviour in physiological measurements. Large-scale datasets could provide the means to establish and validate such models and could ultimately help establishing adaptive DBS paradigms, closing the loop.

## Conclusions

Neither DBS targeting nor stimulation schemes have changed substantially over the last 25 years. To improve DBS therapy and targeting of specific volumes, it will be important to further understand the functional importance of different target areas as well as their structural connectivity and involvement in the genesis of behaviour and clinical symptoms. Finding neural biomarkers for different disease symptoms, as well as the description of their functional involvement during behaviour, will be critical for the further development of closed-loop DBS paradigms. To establish such paradigms, data analysis models that can learn to extract and relate individual neural features to individual clinical symptoms have to be developed and validated. A large-scale comprehensive and standardized dataset incorporating behavioural, as well as neural measurements, could help in developing such models.
